# Greening procurement: Exploring evolving practices in an Irish university

**DOI:** 10.1016/j.heliyon.2023.e21787

**Published:** 2023-11-02

**Authors:** Alexandra Revez, Maria Kirrane, Fiona Thomson

**Affiliations:** a MaREI Centre, Environmental Research Institute University College Cork. Ellen Hutchins Building, University College Cork, Lee Road, Cork, T23 XE10, Ireland; b Office of Sustainability and Climate Action, President's Office, Ground Floor, Hub, University College Cork, College Road, Cork, T12 K8AF, Ireland; c Procurement and Contracts Office, University College Cork, College Rd, 6 Elderwood, Cork, T45 VH39, Ireland

**Keywords:** Green public procurement, Practitioner reflection, Higher education institutions, Horizon scanning, Ireland

## Abstract

Including green criteria in the public procurement of goods and services requires increased expertise, new methodologies, more significant monitoring efforts and more support towards innovation. These added complexities influence procurement professionals and their everyday practices. This article explores the under-researched issue of practitioner-led beliefs, attitudes, and their accounts of Green Public Procurement (GPP). We delve into a qualitative case study of University College Cork (UCC) in Ireland to explore the journey of procurement professionals in introducing GPP across the various sectors and departments of the university. We draw from interviews, a horizon scanning workshop, and secondary materials to capture and build on the expertise of a broad range of staff in UCC with experience in this area. We use this collective viewpoint to make sense of GPP and to position such views relative to ongoing policy priorities, looking at past, present, and future outlooks. The research shows that efforts have been made to introduce green criteria in new tenders. These gradually became wider opportunities to develop competencies, skills, and stimuli to implement more impactful strategies. The research also shows underdeveloped practices around supporting innovation, monitoring, and post-award evaluation. Overall, the paper offers a unique perspective based on the day-to-day practice of public procurement practitioners. While the case study is geographically bound and therefore presents difficulties in replicating findings, it provides a new lens for researching GPP adoption through interaction with practitioners.

## Highlights

• Tracing the greening of procurement as an evolving practice.
• Drawing practical insights from a case study of green public procurement in an Irish University.
• Offering cross-sectoral insights into implementing green procurement in public institutions.
• Adopting a future-oriented approach to identify long-term goals and challenges for GPP practitioners.

## 1 Introduction

Green public procurement (GPP) is increasingly identified as a critical mechanism to influence markets towards more sustainable solutions [[1], [2], [3]]. GPP represents a shift from traditional procurement regimes that have always prioritised value-for-money to one that considers environmental and social criteria [4,5]. This shift from a narrow economic focus to a broader value system based on environmental and socio-economic principles is a critical turning point and a game-changer in public procurement. Yet, as calls seeking to accelerate GPP are becoming more pronounced, there is a danger in assuming it will simply fast-track public institutions to a clearly defined green end goal and direct markets to green practices [6]. This is a problematic assumption because GPP is arguably an incomplete and evolving concept that will continue to grow as new methodologies, empirical evidence, and innovations emerge [5]. Cheng et al. [7] state that GPP is still in its infancy, and more insights are needed to establish ideal institutional dynamics that can support and implement green innovation. In this paper, we aim to revisit the merits, challenges, and outcomes of GPP through the lens of practitioner reflections. Based on an empirical study with procurement practitioners at University College Cork (UCC) in Ireland, we investigated the day-to-day journey of implementing GPP in a public Higher Education Institution (HEI). With increased calls to accelerate and make GPP mandatory across Europe, we stress the value of bringing to the fore the carrying out of ‘real practice’ to understand the changing role of public procurers. This perspective remains largely unexplored, and we hope to provide rich and valuable insights not commonly considered in the GPP literature. We believe this can be a fruitful avenue for future research, and we further propose that the practitioner-reflective approach brings a more dynamic and integrated view of people and institutions.

As such, we consider it more beneficial to understand GPP as a process; as noted by Cousin et al. [8] “[…] ‘greening’ rather than ‘greenness’-could be said to deliver sustainability.” (p.199). This view of GPP draws attention to evolving practice, cumulative effects, and capacity building as critical drivers of sustainability. Such an approach is a good reminder not to take GPP as an effective policy for granted, nor to view it as a simple solution to a complex problem. It stresses the need for a well-grounded take on daily procurement practices, which are neither neat nor fully resolved, but account for the long journey in greening the procurement practices of public institutions. Thus, debates over the best policy approaches towards GPP are ongoing, particularly regarding aligning broader sustainability objectives with green procurement goals [9].

This study uses a mixed-method approach to explore evolving purchasing practices in UCC. We draw on interviews with procurement staff and a horizon-scanning workshop with multiple stakeholders to analyse trends and expectations in the longer term regarding GPP. We look at past, present, and future issues to outline the lessons learned, unresolved challenges, and knowledge gaps within public procurement practice. The paper, therefore, frames GPP as an evolving concept currently developed in the context of uncertainty and volatility regarding the future [10]. This area of GPP research has yet to gain attention. We consider how a practitioner engagement process focused on tracing and anticipating emerging issues may offer new horizons to make GPP more impactful and resilient in the face of growing complexity and disruption. We expect that the insights gained from this approach can help guide the decision-making of institutions and individuals involved in GPP, who must navigate a constantly evolving landscape of interactions.

## 2 Literature review

### 2.1 Beyond value-for-money? – highlights from emerging GPP literature

Research on greening procurement shows that ideas and concepts related to GPP are evolving rapidly in response to increased calls for change and acceleration of GPP practices. This growing body of literature has contributed significantly to key GPP areas such as developing green criteria, green tenders, strategy and management, operational tools, and green legislation. The research includes literature reviews, surveys, desk studies, case studies and theory-driven analyses. Relevant theoretical insights have emerged and include leveraging Organisational Learning Theory to explore experiences of task performance within an organisation and how they are converted into future experience and learning [11,12]; Behavioural and Experimental Economics looking at processes of supplier selection in GPP [13]; Institutional Theory considering the impact of external power on organisational structures, and Upper Echelons Theory, tracing the influential role of top management in driving GPP [2]. Insights associated with the relevance of innovative tools and pilot programmes in mobilising change are also relevant. From a higher education perspective, for instance previous work shows that Sustainability Reporting in HEI's is not widely practised and that increasing Sustainability Reporting could lead to follow-up mechanisms to internalise and improve sustainability [14]. Research also shows that self-reporting tools such as the Sustainability Tracking, Assessment & Rating System (STARS) also provide the means to increase engagement with campus stakeholders [15]. Furthermore, pilot programmes such as the Green Campus have been shown to enhance climate and sustainability literacy for students and staff, with benefits extending through the wider community [11]. However, we find gaps in the literature, namely limitations in the number of qualitative case studies that explore beliefs, awareness, and practices from a practitioner's perspective (ibid).

Commonly, theoretical frames and research findings are directed at practitioners, but few engage qualitatively with the day-to-day context in which GPP practice occurs. Our highlights from emerging GPP literature summarise the current state of the art, and we examine these in the context of practitioners’ changing roles. We situate the article within the theoretical framework of reflective practice and action research to explore everyday dynamics and their potential for promoting change [16].

### 2.2 GPP definition: A new role of public procurement practitioners

GPP broadly refers to the inclusion of environmental criteria in purchasing external goods and services by a public organisation [17]. It is often regarded as a leading policy instrument for reaching broader environmental quality objectives [6,17,18]. It refers to how environmental criteria are incorporated into various purchasing processes, including tendering, selection, evaluation and management of a supply base [19]. One of the most frequent definitions used is that advanced by the European Commission as: “*a process whereby public authorities seek to procure goods, services and works with a reduced environmental impact throughout their life cycle when compared to goods, services and works with the same primary function that would otherwise be procured*.” [20].

More recently, GPP concepts have evolved to embrace ideas around sustainability and circular economy [5]. These emerging strategies highlight the importance of more holistic approaches [5,12]. New terminologies reflecting this shift are used, such as Sustainable Public Procurement (SPP), looking to encompass the ‘triple bottom line’ by advancing approaches that consider in tandem societal, economic and environmental impacts into the purchasing of goods and services [19,21]. Recently, we have seen an alignment between green procurement and circular economy ideals [5], with the European Commission advancing a definition for Circular Public Procurement (CPP) as “*the process by which public authorities purchase works, goods or services that seek to contribute to closed energy and material loops within supply chains, whilst minimising, and in the best case avoiding, negative environmental impacts and waste creation across their whole life-cycle*” [22].

Introducing new concepts and terminologies highlights difficulties in operationalising greening processes in procurement in a way that addresses core sustainability and climate action issues [9]. A recent study accounting for different terminologies stresses the need to use these tools strategically so they can handle a variety of concerns ranging from environmental and social issues to efficiency, animal welfare, anti-corruption and competition [23]. Research on beliefs and GPP practices highlights the value of ‘change agents’ and ‘initiators’ [24,25], and they also emphasise the importance of top executive professionals and their role as transformational leaders to help implement GPP on the ground [26]. There remains a dearth of literature examining the context within which practice occurs, triggers driving action on the ground, and lessons learned from practice-based approaches to change.

### 2.3 Setting GPP criteria: what drives the process?

In Europe, requirements to satisfy GPP have been developed by the European Commission [27]. These requirements are commonly divided into minimum and award compliance criteria [17]. Minimum compliance determines the minimum levels of environmental performance expected of bidders to qualify in the initial selection process; examples include a selection of tenderers with the required certification and those who have shown the ability to apply environmental measures. It also includes meeting technical or functional specifications, such as limits on GHG emissions, performance metrics, and specific requirements, such as limits on the type of fuel used [7,17]. Minimum compliance criteria apply to the qualification stage, where purchasers first select candidates on a pass/fail basis [28]. The next stage is where award criteria apply, where bidders propose innovative solutions and set their strategy. This may include the identification of problems and the development of suitable alternatives [28,29]. A scoring process applies, and environmental criteria are weighed alongside other criteria, such as price [17]. Minimum compliance is the most leveraged set of criteria. Still, it has been noted that minimum compliance is weak as an incentive, offering no pathway for the diffusion of new technologies or products, thus potentially discouraging innovation [7]. It has been argued that it supports short-term thinking that prevents suppliers from being more ambitious about sustainability, providing no consistent pathway or clear-cut direction towards higher standards [9].

Accounting for the growing use of circular economy principles, there is rising interest in adopting life cycle assessment and costing methodologies to establish award criteria holistically, sustainably, and resource-effectively [5,19]. Indeed, Testa et al. [19] have argued that there are synergetic effects in aligning GPP and life cycle costing, seeing that GPP has a strong environmental dimension and life cycle costing has a strong economic dimension. Thus far, there is a lack of clarity on the further integration of social life cycle considerations and the opportunity to assess procurement's human and societal impacts [30].

Consolidation of GPP as a concept and a tool in the pursuit of environmental policy goals at the EU level has been sought through several reforms, the EU Directive concerning public procurement (2014/24/EU) and the EU Utilities Directive (2014/25/EU) seek to provide for better integration of environmental considerations at various stages of public procurement procedures [23], and includes a horizontal stipulation allowing for environmental requirements in procurement, use of criteria underlying environmental labels, accounting for environmental factors across the production process and reinforcing the use of life-cycle analysis into public procurement practices [31]. The EU has further developed and regularly renewed a list of GPP criteria for critical sectors and priority products. However, it premises the use of these criteria as non-binding and subject to procuring authorities’ needs, level of ambition and requirements [32].

More recently, the EU Green Deal [33], which lays out the new EU growth strategy, confirms the ongoing focus on environmental integration and the need for mandatory minimum criteria for GPP [23,34]. It also proposes the phase-in of compulsory reporting to monitor the uptake of GPP and reinforce circular economy principles for future growth strategies and development [34].

Most of the research on green criteria is premised around information deficit models, which propose that training, information sharing, and dissemination of best practices are the main drivers for higher uptake of GPP practices [19,[35], [36], [37]]. However, less is known about practitioners' beliefs, attitudes, and experiences concerning sustainability and how these may provide the motivation and drive for new opportunities for GPP implementation.

### 2.4 GPP variation: Differences in practices across countries and institutions

Despite growing calls to introduce environmental criteria when tendering or awarding contracts and the emergence of promising methodologies, research shows the linear economic approach based on “take-make-dispose” [38] still prevails, with the purchase price, technical considerations, quality and delivery time often outperforming environmental criteria in the decision-making process [28,39,40]. Assessing the uptake of GPP reveals further discrepancies. Current research suggests the uptake of GPP across different European countries is varied. Estimates from 2011 indicate that the lower purchase price criterion remained decisive for 64 % of respondents. As many as twelve countries in the EU27 feature a GPP uptake below 20 % for the ten priority sectors assessed^1^ [41]. This discrepancy is exacerbated by difficulties in determining the gap between high-performing and low-performing countries, as there are currently no monitoring mechanisms to systematically review the performance and uptake of GPP across the EU [4].

Another way variation of GPP uptake is expressed is through institutional aspects. European research suggests smaller, less-resourced public authorities face more barriers to implementing GPP [42]. A mixed-method study from Norway sees a strong correlation between the size of municipalities and the delivery of GPP, which is further correlated with other aspects, such as the existence of a purchasing department and a purchasing strategy [43]. A later mixed method study from Italy by Testa et al. [1] also finds that larger administrations outperform small authorities as the latter face higher barriers in developing GPP initiatives. In this instance, barriers are premised around difficulties defining the appropriate responsibilities, roles, and functions within small administrations to deal with GPP. Results from a large survey of public procurement practitioners across 300 public authorities in the US challenge the idea that larger public organisations perform better. The survey results show substantial variability across and within, different levels of government [44]. The authors in this study argue that green procurement in the US appears instead to be “the result of random and very cautious experimentation with little systematic pattern” to green procurement adoption (p.312) [44].

Variability associated with leadership also appears relevant; Roman [26] has argued that strong GPP uptake and meaningful sustainability actions within institutions are positively tied to leadership styles. Particularly a form of leadership that recognises that the challenges associated with sustainability are not merely technical but also involve human elements.

Differences in GPP uptake across product groups are another relevant factor. Testa et al. [45] identified considerable differences in the performance of Italian institutions among various product groups. Top sector performers include Office IT Equipment, Furniture and Paper. Poor sector performers include Cleaning Services and Products and Construction. A review by Renda et al. [41] among the EU27 also finds variability across product groups; Transport is a top-performing category, whereas Catering, Textiles, Cleaning Services and Products, and Construction trail behind.

### 2.5 Effectiveness of GPP as an environmental policy instrument: An unresolved problem

Arguments supporting GPP are clear and directly link public institutions’ buying power and influence over the market [5,27,46]. In Europe, public purchasing represents a total expenditure of over 2 trillion euros annually [47]. This large market share offers a suitable means to stimulate eco-innovations, ensure standards are met, and reduce the impact of public services and activities on the environment [1,4,12]. Yet, evidence reveals that GPP needs refining and more strategic thinking as an environmental policy instrument to ensure consistency [6,7,48]. The underlying assumption is that GPP will enlarge the market for more sustainable products and services, working as a trigger or signal for suppliers [6]. However, it can often occur that GPP displaces other consumers out of the green market and into the brown market (ibid). Recent research also suggests that GPP is more likely to trigger innovation in smaller and medium-sized firms than in larger ones [49]. Furthermore, the cost of meeting certain environmental standards may be a disincentive to producers and suppliers, particularly if they require substantial adjustments to meet new standards [6].

Additionally, there is a lack of robust empirical evidence across Europe to establish the effectiveness of GPP. Cheng et al. [7] argue there is a lack of vision of how GPP policy affects the market, how it affects consumer demand for green products, and what are its long-term sustainability goals. Lundberg, Marklund and Strömbäck [6] provide a critique of GPP as a policy instrument and show its effectiveness to depend on several factors, including product characteristics, market power, and price sensitivities of private and public consumers. Challenges to GPP suggest that other policy instruments, such as direct subsidies to producers or taxation in certain contexts or for specific sectors, can be more impactful [6,48].

Yet despite the current lack of evidence around the effectiveness of GPP, counterarguments point to the leading role of public procurement in achieving social innovation and delivering positive policy goals. Notably, the work of McCrudden [50] tracing the use of government contracting across the US and beyond shows how it has historically been used to address various social issues such as labour standards, unemployment, human rights, and equality around gender, ethnicity and expanding opportunities for disabled workers. Research from Germany showcases the value of GPP as an eco-innovation instrument, albeit these findings stress that innovations of a more incremental character and that bear lower implementation risk, are preferred by procurers [51].

### 2.6 GPP policy in Ireland: Situating the current policy and practice context

The sections above frame the current state of play concerning GPP practice in Ireland. The Irish *Circular Economy Programme* (2021–2027) and the *Green Public Procurement Guidance for the Public Sector* [[52], [53]] outline the approach adopted so far, primarily left to the discretion of the various state departments and public bodies.

There has been a growing interest in GPP's role in meeting current climate change and circular economy ambitions, as seen in Table 1. Like other GPP policy trajectories in Europe, we have seen a closer alignment between GPP and circularity. Equally, in parallel to calls across Europe to see GPP progress from non-binding commitments to mandatory uptake, we note that in the most recent Green Public Procurement Guidance for the Public Sector (2021), there is a stipulation to make GPP mandatory for all procurement using public funds by 2023. This, however, does not appear to be reinforced in other key policy documents, such as the most recent *Climate Action Plan CAP23* [54].

**Table 1:** Timeline of GPP policy developments in Ireland.

Timeline	Policy and Guidance	GPP Goals
	Climate Action Plan CAP23	Review Green Tenders and develop a new GPP strategy, zero-emission vehicles, from Jan 1st, 2023.
	EPA Strategic Plan	The strategic goal for all public procurement is to factor in reduced environmental impacts through GPP by 2026.
2022	GPP Criteria Search	Launch of the online search tool to find Irish GPP criteria.
	The Circular Economy Programme	Preventing waste and driving the circular economy, identification of GPP as a pillar for Innovation and Demonstration.
	Green Public Procurement Guidance for the Public Sector	Update from 2014 guidance on ten criteria sets and twelve products/services: advice on monitoring and reporting on GPP, statement of Ireland's commitment to implementing GPP in all tenders by 2023.
2021	Climate Action Plan 2021: Securing Our Future	Commitment to publish a whole-of-government Circular Economy Strategy; requirement for semi-state bodies to promote circular economy measures and GPP in all procurements using public funds; broadening the scope of GPP across different sectors such as transport, housing, and ITC.
	Strategic Procurement Advisory Group	Call to mobilise expertise from critical bodies in the environmental sphere.
	Local Authority Climate Charter	Committing to a GPP strategy across all business sectors; requesting carbon footprint information from suppliers.
2019	Climate Action Plan 2019: To Tackle Climate Breakdown	A phased introduction of GPP; specified public transport fleet frameworks and building criteria; delivering a GPP approach which incorporates carbon pricing and climate criteria
2012	Green Tenders	The first GPP action plan introduced in Ireland calls for public bodies to progressively integrate green criteria into public sector tendering.

Academic literature relevant to Ireland regarding GPP policy and performance is limited. Estimates from 2011 assessing the uptake of GPP in the EU27 place Ireland within the lower-performing countries, noting Irish GPP uptake was below 20 % for the ten priority sectors considered. Gormly [55] looks at the Irish context in more detail. The article draws from interviews with practitioners and identifies barriers to GPP. These include a lack of consistency regarding working definitions of sustainable procurement, absence of mandatory guidelines, issues around added cost and time, and lack of knowledge by suppliers.

A monitoring report on GPP activity in Ireland was recently submitted by the Environmental Protection Agency [56]; it finds a low level of implementation of GPP across government departments. Results show that including GPP criteria in contracts over €25,000 represents an average of 17 % of total spending. The highest reported spend came from IT Equipment, and the lowest was on Textiles. There was also considerable variability between departments, ranging from 0 % to 100 %, concerning their reported spending on contracts containing GPP criteria. The report notes that this variability is partially a result of a lack of internal data retention or the fact that the available data is incomplete.

## 3 Methodology: A case study exploring GPP at university college cork

### 3.1 Research approach

In the social sciences sphere, it has long been argued that we must reframe our methodologies and assumptions commonly grounded on information scarcity and knowledge deficits and advance a reflexive research approach that embraces situated and emergent ways of knowing [57]. We argue, therefore, that the research challenge that we face nowadays is not necessarily to address solely information shortfalls but to offer timely and meaningful information in the context of ‘super-abundance’ [58,59] and accelerated circulation of knowledge [60]. In this paper, we aim to revisit the merits, challenges, and outcomes of GPP through the lens of practitioner reflections. Based on an empirical study carried out with procurement practitioners in UCC in Ireland, we propose investigating the day-to-day journey of implementing GPP in a public HEI setting. With increased calls to accelerate and make mandatory GPP across Europe, we stress the importance of bringing to the fore the carrying out of ‘real practice’ to understand the changing role of public procurers and the challenges they face. This perspective remains largely unexplored, and we hope to provide rich insights not commonly considered in the GPP literature. To the best of our knowledge, practitioner reflections have not been leveraged within this body of research. Specifically, we build on the concrete case of UCC as an opportunity to co-create new knowledge and bridge ‘official’ accounts of practice with the ‘thicker’ and context-rich versions of public procurement practitioners. We emphasise the productive aspects of practitioner-led reflections as theories-in-the-making arising from the everyday application of concepts, learning by doing, and situated views on future outlooks [[61], [62], [63]].

### 3.2 Data collection

We followed a three-stage approach focused on the qualitative case study of greening practices at University College Cork (UCC), which involved various data collection activities (see Table 2). Using case studies is a well-established strategy seeking to deepen insights through complementary data sources that combined provide for a contextually rich and diverse perspective. While case-study insights are context-dependent and specific to ‘a case’ [64], they are essential building blocks for learning and building know-how. The value of qualitative case studies is their opportunity for in-depth analysis of details and nuances often overlooked by other data collection methods. Replicability and multiplying effects associated with case studies rely on deeper analysis and the generalisation of findings that may be applicable elsewhere. A recent systematic literature review of GPP by Cheng, Appolloni [7] identify several case studies exploring GPP, but they highlight a dearth of in-depth qualitative materials. Previous case studies include sectoral-specific research such as the work of Aldenius and Khan [17] exploring the strategic use of GPP in the bus sector; the work of Alvarez and Rubio [47] exploring GPP pathways to account for the carbon footprint within the services sector; research by Brindley and Oxborrow [65] exploring a UK university catering department; and a study by Hupponen, Grönman and Horttanainen [66] focused on solid waste management procurement.

**Table 2:** Data collection activities.

Phase	Focus	Methods
1	Exploring past experiences of GPP and tracing early interventions	Desk Research Interviews
2	Current approaches and present views of green procurement	Desk Research Interviews
3	Future outlook	Interviews Horizon Scanning

Our case study offers a broad perspective drawing from qualitative insights into GPP in the university. The three-phase approach looked at past, present, and future insights to trace how GPP and ‘greening’ practices continue to evolve within UCC. Data collection included interviews, a horizon scanning workshop and a review of the university's desk materials linked to GPP. These data collection methods offer a flexible and open approach to engaging with the journey undertaken in UCC to introduce GPP and the experiences and practices of the professionals in the process. Table 2 shows that the various methods allow for a different focus and bring a temporal element to tracing this journey.

A review of secondary data sources comprised key materials, including *UCC Sustainability Strategy* [67], *UCC Strategic Plan 2017–2022* [68], and *UCC 2022 Delivering a Connected University* [69]. Interviews entailed a semi-structured script and an interventionist interview that sought to involve participants in problem-solving where pertinent [70]. We carried out six online interviews (four male and two female participants) with procurement staff at UCC. We used purposive sampling, leveraging the knowledge of key staff concerning GPP practices to choose a representative sample [71]. These are staff deemed to have extensive, relevant, and varied experiences concerning GPP in the university. A list of key GPP practitioner sectors was compiled to facilitate selection and sampling frames, including Catering, Buildings and Estates, IT, Cleaning, Labs and Energy. The selection process began with identifying representative participants within one or more sectors.

We conducted an online horizon scanning (HS) workshop with fifteen participants (eight male and seven female). Using purposive sampling, the workshop included those with expertise or experience in procurement within UCC and a student representative. HS is a sense-making tool that connects meaningfully with modern society's increased complexity and uncertainty by adopting forecasting tools beyond linear extrapolation or casual modelling [72]. It aims to identify emergent issues that may present as threats or opportunities for society [73]. The process triggers knowledge creation through a time-bound identification of issues, followed by prioritisation and development of recommendations [74].

The online workshop was 90 min long. The process opened with a guest speaker presentation from the EPA who provided an overview of GPP policy developments in Ireland. The HS exercise was premised on six interactive questions, as shown in Table 3. The results of each question appeared live on screen, and participants engaged and commented on the results in real-time.

**Table 3:** Horizon Scanning interactive questions.

Number	Question
1	What are the key drivers in your purchasing decisions?
2	In the short term, what changes do you foresee in GPP (1 year)?
3	In the medium term, what changes do you foresee in GPP (5 years)?
4	In the Long term, what changes do you foresee in GPP (10 years)?
5	What key opportunities should be catalysed, and what key issues mitigated? (*prioritisation*)
6	This prioritisation reflects my views?

Ethical approval for this study was obtained from the Social Research Ethics Committee at UCC. All participants were informed of the key objectives of the study and its academic purpose. The voluntary and confidential principles underpinning the research process were outlined, and consent was obtained before data collection.

### 3.3 Data analysis

We used thematic analysis to process, analyse and structure our findings to make sense of the data collected. Thematic analysis is a data analysis technique which enables the researcher to identify patterns and themes in the data set. Subsequently, it provides categories for analysing and reporting this information [75]. We used NVivo to support the thematic analysis. The initial thematic analysis was performed by one researcher, where an independent exploratory analysis was carried out with no predefined structure. Themes were identified and coded as they emerged in the various materials. This was subsequently refined with the feedback of the wider research team. A content analysis of the literature was also performed, exploring clusters of themes associated with the perception of change for the short, medium, and long terms. The NVivo software effectively allows open coding and refining the thematic process by identifying relevant sub-themes and exploring relationships between themes [76,77].

## 4 Findings and discussion points (past, present and future insights)

### 4.1 Past experiences and triggers for sustainability

Sustainability came into prominence at UCC over fifteen years ago with the start of the Green Campus Programme. This was an Eco-Schools initiative led by students with the help of *A Taisce* (the National Trust for Ireland). The Eco-schools programme began at primary and secondary levels in the 1990s. It promoted student leadership and activism for creating more sustainable school environments, recognised by awarding schools a Green Flag. In 2007, students looked to replicate the programme at UCC. Their efforts were rewarded, and 10.13039/501100001636UCC became the first university in the world to be awarded a Green Flag. This voluntary programme has expanded over the years, and nearly all primary, secondary, and third-level institutions in Ireland now have a Green Flag. Based on ISO14001, the addition to the programme is that it must be linked to the curriculum. Equally, the programme must have buy-in from all parts of the university. This gave rise to UCC's approach, expressed as being *student-led, research-informed and practice-focused*. The reach and scope of UCC's sustainability strategies have increased rapidly and include sustainable procurement in various priority sectors and divestment of its Trust from fossil fuels; currently, 70 % of UCC's trust is invested in ‘positive sustainability investments’ [78].

This trajectory is not surprising; in the 90s and early 00s, environmental concerns rarely featured as an issue for the procurement office. Standards such as the ISO 14000 changed this (much like the ISO 9000 quality standards in the 70s and 80s), and they became widely accepted by businesses to assess environmental footprints and as promotional points [8]. UCC's trajectory also parallels trajectories in other universities, such as the University of Turin, which traces its first steps into sustainability back to 2006 [79].

A key point in tracing earlier experiences of GPP emerging from the interviews is that early efforts were employed on an ad-hoc basis, with limited integration into operations management strategies. This more casual engagement with GPP was apparent in the bidding process:

“We're not putting [GPP] in as a token gesture anymore, which might have been the case five or six years ago. [Bidders] would send sustainability policy that they drafted on Word 60 minutes before submitting the tender […] We are going a lot further than that now.”

In hindsight, such unplanned approaches may seem to have little substance and were tokenistic. Yet, the build-up of these experiences may have provided new opportunities to gradually develop competencies, skills and stimuli to implement more impactful strategies [1]. They may also express what Prier et al. (2016) see in early procurement processes in the US as random and cautious experimentation.

During the interviews, various triggers are mentioned as relevant. These encompass factors that instigated, motivated, or provided an opportunity for introducing more strategic responses to greening procurement. These triggers include, in order of prominence.

• Support from decision-makers and procuring staff.
• The ‘Green Campus’ Programme
• Personal interest in sustainability
• The introduction of the sustainable print management policy
• The UCC ‘Saver Saves’ Scheme [80].

The first two points broadly refer to a strong recognition of the legacy of sustainability practice in UCC, found to be encouraging and compelling. Ownership and pride in this legacy are relevant triggers.

“Sustainability has been a strategic direction of the university for years and years, and it's one of the top universities in the world for sustainability. So that's very helpful in making sure that the cultural change is as pain-free as possible”.

The third trigger refers to personal interest and environmental mindedness, identified as a big motivating factor to initiate workplace change. The fourth factor refers to sustainable print management policy, a university-wide measure introduced in 2019 to replace individual printing systems with a shared and centrally managed print system [81]. Finally, the UCC “Saver Saves” Scheme targeted the top 13 buildings (out of a total of 130 buildings) that consumed 87 % of the university's energy [80]. The scheme aims to reduce energy use within these buildings while allowing financial savings to remain with the department/school to reinvest in further environmental projects.

### 4.2 Current GGP practices and managing change

**Fig. 1:**
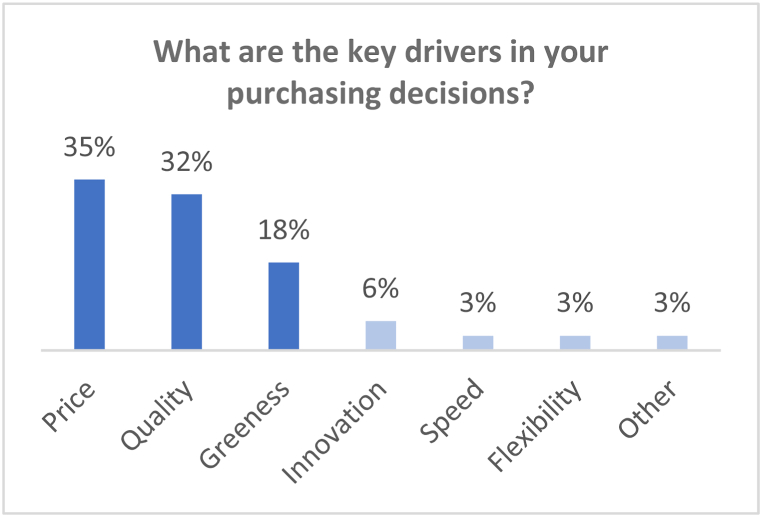
Results of online HS workshop- Question 1.

It is only when we look back at the last fifteen years in UCC that we can recognise the shift that has taken place in procurement practices from cost-based decisions on purchases to multifaceted sustainability decisions on purchases. Yet, this is not a straightforward change, and critical factors such as price and product quality continue to dominate the decision-making process. As shown in Fig. 1, drawing from the workshop results, ‘greenness’ occupies third place as a driver in purchasing decisions. If we align the workshop results with interview data, we can start to qualify these shifts as they emerge in current practices and decision-making processes.

All interview participants recognise that UCC is in a transition phase, which has changed how procurement is managed.

“I think we're moving to a phase of actually, you know […] this is something that we do. It’s a strand in everything that we're doing now”.

One exciting aspect of this transition phase concerns the approach taken to manage change. This aligns with the Green Campus programme founded on cooperation, consensus and continual improvement. These cooperative structures influence how GPP has been introduced and embedded in UCC as a gradual and transitional process. Equally, the Irish policy approach to GPP has also focused on a phased introduction of green criteria, influencing the approach adopted [82]. Lack of confrontation or drastic change until now is seen in a positive light with some notable examples across the university, such as the farm-to-fork initiative, the managed print services, and the elimination of single-use plastics, all done in a phased manner. The example offered by one of the interview participants illustrates this approach well:

“They [another University] implemented their managed print service. They actually went for a big-bang approach. They basically got rid of everyone's desktop printer in the space of a couple of weeks […], and someone had actually chained their printer to the desk […] Our approach at UCC has been a little bit more transitional, a more incremental approach to it rather than a big bang [ …] So we avoided those confrontations, which I think I suppose is a good thing.”

Other participants further qualify the introduction of gradual change as necessarily a *‘pragmatic approach’* that accounts for the complexity of the university system—for instance, recognising that putting in place innovative technologies (while desirable) may be incompatible with some systems and equipment used by specific departments for specialised purposes. Drastic change, in this case, would be counterproductive if, as a result, incompatible equipment had to be replaced. In this instance, interoperability is advanced as an area requiring attention, particularly around IT equipment. Also, there is a need to ascertain whether new practices or systems compromise certain protocols, such as laboratory conditions and processes.

Another aspect linked to the complexity of the university system relates to a devolved governance structure. This means the various departments across the university have control and responsibility over their purchases. These would be smaller purchases (under €25, 000) that don't require a tender process. Altogether, they amount to many purchases beyond the control of the procurement office. This is perceived as a blind spot by some of the participants and considered in some ways as an easy win in terms of greening procurement opportunities when compared to other initiatives, such as installing new energy-efficient equipment or retrofitting buildings that entail substantial up-front costs. Changes identified at this level include introducing GPP criteria, reducing the use of resources where possible, and sharing resources across different departments. Introducing stricter GPP protocols to research funding processes is another important step to direct single-purpose purchases towards greener processes.

Finally, workshop results, as per Table 4, suggest work practices must continue to change across UCC. 39 % of changes perceived in the short term refer to work practices and include various issues, some of which are practical issues relating to the need for more training for staff and suppliers, introducing new methodologies, more monitoring and reporting, and dealing with uncertainty and risk. This area was identified as a priority in the short term. Further, recommendations included opt-in programmes to educate suppliers, better communication, and simple protocols for tracking supplier performance.

**Table 4:** HS workshop. Changes foreseen in the short, medium, and long-term regarding GPP. Emerging findings are organised into clusters, and each dot represents an entry point.

		TIME HORIZON ENTRIES		
		Short term *1 Year.*	Medium Term *5 Years.*	Long Term *10 Years.*
**CLUSTERS**	Carbon Emissions		**●●●●**	
	Circularity		**●●●**	**●●●●**
	Economy			**●●**
	Energy	**●●●**	**●●**	**●●●**
	Environment			**●●●●●**
	Geopolitics	**●●**		
	GPP Criteria	**●●●●●●●●**		
	Supply Chain	**●●●**	**●●●●●**	**●●●**
	Technology			**●●●●●**
	Transport		**●●**	
	Work Practices	**●●●●●●●●●●●●**	**●●●●●**	**●●●●**

There is a more critical aspect regarding work practice issues, which broadly refers to a perceived culture of exceptionalism that may have allowed a cohort of more established university staff to be impervious to the change processes that newer generations encounter. Furthermore, introducing change in some areas can present added challenges, as outlined in the quote below.

“Printing and parking were the two things that everyone felt that was owed to everyone, you know, and unfortunately, on both counts, sustainability-wise, those things are going to be impinged on.”

### 4.3 Future insights

Medium to long-term issues include the widespread use of carbon budgets, carbon footprints, and supply chain life cycle assessments, with the expectation that these will play a significant role in how UCC assess and manages sustainable procurement (see Table 4). Circularity is another important and interlinked factor identified during the interviews and the workshop, where reuse, repair and recycling of materials are discussed.

Carbon Footprint (CF) is a key environmental indicator. CF in the literature is proposed as a way to assess changes in consumption in a comprehensive manner [47]. However, downscaling carbon budgets at the sub-national level, and assessing carbon footprints present methodological challenges, including the scope of emissions, whether to account for carbon off-setting and removal, geographical attribution of emissions, and the choice of an emission accounting system [83]. Current difficulties include the need to develop a consistent and transparent approach that measures CF along the supply chain and follows the lifecycle of individual products. The time and expertise required to do this are barriers to consolidating these practices in the coming years [5]. These concerns parallel those expressed at the workshop.

Issues around circularity in UCC include ongoing efforts to understand how waste is managed and how green or recycled materials become spoiled or rejected. Another concern is the right to repair and bypass built-in redundancies [34]. Right-to-repair compels manufacturers to design sustainable, long-lasting products that can be repurposed at the end of their lives. Workshop recommendations include developing and supporting supplier repair practices and take-back arrangements. Overall, interviewees welcome the likelihood that GPP may become mandatory, but are concerned about whether it will lead to price inflation and have a minor impact on the regular market in terms of spearheading innovation and sustainability.

“Government needs to make big decisions about … [making GPP mandatory], but if they're going to do it and they're going to impose it only on the public sector, the danger is that you're going to kind of end up with suppliers gouging the public sector for profit. […]it just ends up costing the public sector a fortune. […] but it doesn't really change any practices, and it doesn't really have any sort of a long-term benefit because you're making the public sector do something, and there's a whole world of private out here doing their own thing”.

Energy is mentioned across all time horizons during the workshop. Energy efficiency, energy autonomy, and cleaner energy solutions emerge as goals for the long term. Energy Performance Contracting (EPC) and Energy-*as*-a-service model are discussed during the interviews; these are seen as options to improve building energy efficiency. However, based on previous experiences with the process, they were found to be risky, given they are time-consuming and render limited results in terms of choice of solutions and suppliers. Findings from the literature looked at measures to de-risk EPC projects. They found that the provision of project facilitators in the US and the amendment of procurement procedures have seen positive results [84]. The pooling of resources with other big energy users in the city is also mentioned as a promising approach for commuting and energy use opportunities. Issues associated with climate change and environmental impacts emerged as a concern in the long term; they include material shortages, disruption of service provision and worsening weather events.

### 4.4 Practitioner contributions to current GPP debates

The literature on GPP is varied and rich and is growing rapidly. However, directing research findings at practitioners without fully grasping their beliefs, values, and practices is counterproductive. Practitioners are at the coalface of GPP implementation and have valuable granular knowledge needed to recognise issues and priorities for action. Critically, we note from our engagement with practitioners that in the last fifteen years, UCC procurement professionals have seen their role considerably reinvented. Incorporating greening processes into procurement is complex, and their voice is critical in naming, framing, and contextualising the procurement system changes that we strive to achieve to tackle climate change. Ideas around reflection emphasise the importance of learning through reflection on ‘doing’. To help capture some of our key findings, we adapted a framework proposed by Rolfe et al. [85] drawing from Borton's Reflective Practice model [86]. The model provides a simple structure (as per Table 5) that positions different insights within three distinct spheres of ‘reflection’, designated as the Descriptive Level of Reflection (*What? - A problem framing sphere),* Knowledge Building (*So What? - A value framing sphere*) and Action Oriented Reflection (*Now What? - A solution framing sphere*).

**Table 5:** -Summary of findings using the Reflective Practice Model adapted from Borton [86] and Rolfe et al. [841].

Descriptive Level of Reflection	Knowledge Building	Action Oriented Reflection
*What?*	*So what?*	*Now what?*
Experiences with GPP have evolved from ‘ad hoc’ practices to deeper integration into operations management strategies. Various triggers and flagship projects (such as the Green Campus Programme, support from top management and personal interest in sustainability, sustainable print management and the ‘saver saves’ scheme) marked the transition to more strategic GPP practices. Incremental change and engagement with staff and students have provided a non-confrontational approach to GPP. The stance of practitioners over mandatory GPP was uncertain. While mandatory GPP policy was welcome, we noted concerns over market responses to this, such as price hikes in the public sector, lack of quality service and doubts about whether it will lead to considerable changes in the broader market.	Early experiences with GPP reinforce the value of building on a legacy of good practice around engagement, top-level management commitments and the ongoing development of competencies and skills. Drawing and expanding on the successes of flagship projects and celebrating success drives good practice and further innovation. Practices such as reducing, re-purposing, repairing or sharing resources usually do not feature in current considerations for greening procurement. These typically fall outside the remit of purchasing decisions and show that some sustainability measures extend beyond ‘buying’ decisions and require a new form of partnership and alignment with other public service practices and strategies. Relevant questions emerge on managing and accelerating change and moving past current incremental transition approaches to GPP.	A discussion concerning short-term priorities for greening procurement in UCC reveals that more immediate green measures are geared to consumer behaviour change and changes in work practices. Examples include concerns for exploring resource-sharing arrangements and finding ways to maximise the use of existing resources and technologies. Procurement staff also noted that green public procurement is not solely based on the readiness of procurers but also the readiness of suppliers across the supply chain. Clear messaging on what is coming from a supplier perspective is critical to ensure widespread adoption. Carbon footprints and life-cycle assessments are positioned as critical methodologies to advance GPP. Weaknesses to be addressed include practices around monitoring and evaluation capabilities.

## 5 Conclusion

In the last ten years, calls to reduce the environmental impact of public consumption of goods and services through GPP have intensified [23]. Most policy in the European context has been based on non-binding guidelines and left to the discretion of contracting authorities. Current debates over increasing legislative incentives, including mandatory sectoral GPP rules, seek to strengthen the potential and effectiveness of GPP as a critical environmental and sustainability policy measure (ibid). Considering key European policy developments, we provided an overview of how this process has played out in the Irish context. We delve into the case-study of University College Cork in Ireland to examine how it has introduced GPP across the various sectors and departments. We drew from a review of secondary materials, interviews and a horizon scanning workshop to capture and build on the expertise of a broad range of staff in UCC with experience in this area. We used this collective viewpoint based on the expertise of multiple stakeholders across UCC to make sense of GPP and to position such views relative to ongoing policy priorities, looking at past, present, and future outlooks. We premised the notion of GPP tied to evolving terminologies and concepts and stressed the need to focus on ‘greening’ as a process-driven idea. We conclude by emphasising three key points in this regard.

First, while we have found that a transitional approach to greening procurement has been relevant and shown results in introducing and implementing GPP across the university, we suggest that gradual approaches to implementing GPP may not serve all contexts. More transformative approaches may be needed; in such instances, strong leadership is necessary. Roman [26] has shown that, particularly in the context of growing uncertainty, transformational leadership can instigate and steer change processes.

Secondly, commitment to mandatory GPP in Ireland through policy appears equivocal and vague. Equally, the stance of UCC staff members over mandatory GPP also seems uncertain. While interview participants welcome mandatory GPP policy, they also state concerns over market responses to this, namely concerns over price hikes in the public sector leading to no considerable changes in the broader market structures. However, the role of GPP to promote innovation and demonstrate more sustainable practices is promising and should be leveraged in priority areas and sectors such as energy. Furthermore, mandatory GPP is not solely based on procurers' readiness but also on suppliers' readiness. Clear messaging on what is coming ahead is critical to ensure widespread adoption for multiple stakeholders.

Finally, greening procurement in the short to medium term is associated with developing and promoting new working practices. These include adopting and acquiring expertise in using new methodologies such as life-cycle assessments, carbon footprint measurements, and meeting new performance standards. Like findings across Europe, we have found that reporting and monitoring capabilities are particularly limited. Contract performance mechanisms could be a way to improve this issue, including baseline indicators to be monitored over time.

### 5.1 Research limitations and future research

Given the research's geographically bounded context, we acknowledge limitations regarding our findings' replicability. Another limitation associated with practitioner reflections is that the findings are strongly tied to practitioners' ability and willingness to critique their practice. While we have found the practitioners to be open and willing to engage with the research process, there can be limitations or biases in how practitioners appraise their performance [87]. While there are limitations, we argue that the results can be applied elsewhere. Like Ireland, many other European countries have struggled to embrace the potential of GPP fully, and many are only starting to enter this space; thus, learning from the UCC journey as related by the practitioners involved can be informative and enabling. The approach we employed in this paper drawing from practitioner reflections has allowed for a more dynamic view of people and institutions; this has seldom appeared in the literature to date. We further suggest that comparing the UCC journey with other European cases could be fruitful for future research.

## Additional information

No additional information is available for this paper.

## Data availability statement

No additional data is available for this paper. The data that has been used is confidential.

## Funding

This work was supported by 10.13039/501100001636UCC Green Campus and 10.13039/501100001602Science Foundation Ireland through the MaREI Centre for Energy, Climate and Marine. The funders had no role in the design of the study.

## CRediT authorship contribution statement

**Alexandra Revez:** Conceptualization, Data curation, Formal analysis, Project administration, Software, Visualization, Writing – original draft, Writing – review & editing. **Maria Kirrane:** Conceptualization, Formal analysis, Funding acquisition, Investigation, Supervision, Validation, Writing – original draft, Writing – review & editing. **Fiona Thompson:** Conceptualization, Formal analysis, Funding acquisition, Supervision, Validation, Writing – original draft, Writing – review & editing.

## Declaration of competing interest

The authors declare that they have no known competing financial interests or personal relationships that could have appeared to influence the work reported in this paper.

## Footnotes

1 Ten priority sectors considered, based on common EU criteria are: cleaning products and services; construction; electricity; catering services and food products; gardening services and products; office IT equipment; copying and graphic paper; textiles; transport; and furniture. The list has since increased. An updated list is available: https://ec.europa.eu/environment/gpp/eu_gpp_criteria_en.htm.

## References

[1] Testa F. (2012). What factors influence the uptake of GPP (green public procurement) practices? New evidence from an Italian survey. Ecol. Econ..

[2] Ma Y. (2020). Does green public procurement encourage firm's environmental certification practice? The mediation role of top management support. Corp. Soc. Responsib. Environ. Manag..

[3] Lindström H., Lundberg S., Marklund P.-O. (2020). How Green Public Procurement can drive conversion of farmland: an empirical analysis of an organic food policy. Ecol. Econ..

[4] Brusselaers J., Van Huylenbroeck G., Buysse J. (2017). Green public procurement of certified wood: spatial leverage effect and welfare implications. Ecol. Econ..

[5] Sönnichsen S.D., Clement J. (2020). Review of green and sustainable public procurement: towards circular public procurement. J. Clean. Prod..

[6] Lundberg S., Marklund P.-O., Strömbäck E. (2016). Is environmental policy by public procurement effective?. Publ. Finance Rev..

[7] Cheng W. (2018). Green Public Procurement, missing concepts and future trends – a critical review. J. Clean. Prod..

[8] Cousins P., Lamming R., Squire B. (2008). Strategic supply management: principles, theories and practice.

[9] Melissen F., Reinders H. (2012). A reflection on the Dutch sustainable public procurement programme. J. Integr. Environ. Sci..

[10] Christopher M. (2018). The mitigation of risk in resilient supply chains. OECD/ITF joint transport research Centre discussion papers.

[11] Reidy D., Leal Filho W. (2015). Integrative Approaches to Sustainable Development at University Level: Making the Links.

[12] Braulio-Gonzalo M., Bovea M.D. (2020). Criteria analysis of green public procurement in the Spanish furniture sector. J. Clean. Prod..

[13] Igarashi M., de Boer L., Pfuhl G. (2017). Analyzing buyer behavior when selecting green criteria in public procurement. J. Public Procure..

[14] Filho W.L. (2020). Sustainability leadership in higher education institutions: an overview of challenges. Sustainability.

[15] Kirrane M.J., Leal Filho W., Borges de Brito P.R., Frankenberger F. (2020). Trade and Institutional Sustainability.

[16] Leitch R., Day C. (2000). Action research and reflective practice: towards a holistic view. Educ. Action Res..

[17] Aldenius M., Khan J. (2017). Strategic use of green public procurement in the bus sector: challenges and opportunities. J. Clean. Prod..

[18] von Oelreich K., Philp M. (2013). Green Public Procurement: a Tool for Achieving National Environmental Quality Objectives.

[19] De Giacomo M.R. (2019). Does green public procurement lead to life cycle costing (LCC) adoption?. J. Purch. Supply Manag..

[20] (2008). Commission of the European Communities, Communication from the Commission to the European Parliament, the Council, the European Economic and Social Committee and the Committee of the Regions on the Sustainable Consumption and Production and Sustainable Industrial Policy Action Plan.

[21] Cerutti A.K. (2016). Carbon footprint in green public procurement: policy evaluation from a case study in the food sector. Food Pol..

[22] European Commission (2017). Clei - local governments for sustainability. Public procurement for a circular economy: good practice and guidance.

[23] Pouikli K. (2021). Towards mandatory Green Public Procurement (GPP) requirements under the EU Green Deal: reconsidering the role of public procurement as an environmental policy tool. ERA Forum.

[24] Grandia J. (2015). The role of change agents in sustainable public procurement projects. Publ. Money Manag..

[25] Grandia J., Steijn B., Kuipers B. (2015). It is not easy being green: increasing sustainable public procurement behaviour. Innovation: Eur. J. Soc. Sci..

[26] Roman A.V. (2017). Institutionalizing sustainability: a structural equation model of sustainable procurement in US public agencies. J. Clean. Prod..

[27] European Commission (2016). European Union Publication Ofﬁce.

[28] Igarashi M., de Boer L., Michelsen O. (2015). Investigating the anatomy of supplier selection in green public procurement. J. Clean. Prod..

[29] Lindfors A., Ammenberg J. (2021). Using national environmental objectives in green public procurement: method development and application on transport procurement in Sweden. J. Clean. Prod..

[30] Dragos D., Neamtu B. (2014). Sustainable public procurement: life-cycle costing in the new EU directive proposal. Eur Procure Public Priv Partnersh Law Rev.

[31] European Commission (2016). Public Procurement Reform Factsheet No. 7: Green Public Procurement.

[32] European Commission (2022). EU GPP criteria.

[33] European Commission (2019).

[34] European Commission (2020). Circular economy action plan. For a cleaner and more competitive Europe.

[35] Erridge A., Hennigan S. (2012). Sustainable procurement in health and social care in Northern Ireland. Publ. Money Manag..

[36] Preuss L., Walker H. (2011). Psychological barriers in the road to sustainable development: evidence from public sector procurement. Publ. Adm..

[37] Testa F. (2016). Examining green public procurement using content analysis: existing difficulties for procurers and useful recommendations. Environ. Dev. Sustain..

[38] Geisendorf S., Pietrulla F. (2018). The circular economy and circular economic concepts—a literature analysis and redefinition. Thunderbird Int. Bus. Rev..

[39] Braulio-Gonzalo M., Bovea M.D. (2020). Relationship between green public procurement criteria and sustainability assessment tools applied to office buildings. Environ. Impact Assess. Rev..

[40] Fuentes-Bargues J.L., González-Cruz M.C., González-Gaya C. (2017). Environmental criteria in the Spanish public works procurement process. Int. J. Environ. Res. Publ. Health.

[41] Renda (2012). Centre for European Policy Studies (CEPS) and College of Europe: Brussels.

[42] Rosell J. (2021). Getting the green light on green public procurement: macro and meso determinants. J. Clean. Prod..

[43] Michelsen O., de Boer L. (2009). Green procurement in Norway; a survey of practices at the municipal and county level. J. Environ. Manag..

[44] Prier E., Schwerin E., McCue C.P. (2016). Implementation of sustainable public procurement practices and policies: a sorting framework. J. Public Procure..

[45] Testa F. (2016). Drawbacks and opportunities of green public procurement: an effective tool for sustainable production. J. Clean. Prod..

[46] Neubauer C., Policy P.D.f.E.a.S. (2017). Study for the ENVI Committee.

[47] Alvarez S., Rubio A. (2015). Carbon footprint in Green Public Procurement: a case study in the services sector. J. Clean. Prod..

[48] Lundberg S., Marklund P.-O. (2018). Green public procurement and multiple environmental objectives. Econ. Polit. Ind..

[49] Krieger B., Zipperer V. (2022). Does green public procurement trigger environmental innovations?. Res. Pol..

[50] McCrudden C. (2004). Using public procurement to achieve social outcomes. Nat. Resour. Forum.

[51] Czarnitzki D., Hünermund P., Moshgbar N. (2018). ZEW-Centre for European Economic Research Discussion Paper.

[52] EPA (2021).

[53] EPA (2021). Green public procurement guidance for the public sector. Environ. Prot. Agency.

[54] DECC (2022). C.a.C..

[55] Gormly J. (2014). What are the challenges to sustainable procurement in commercial semi-state bodies in Ireland?. J. Public Procure..

[56] EPA (2022). Environmental Protection Agency.

[57] Luker K. (2009).

[58] Kearney R. (1988). The wake of imagination..

[59] Andrejevic M. (2013).

[60] Skjølsvold T.M., Coenen L. (2021). Are rapid and inclusive energy and climate transitions oxymorons? Towards principles of responsible acceleration. Energy Res. Social Sci..

[61] Schön D.A. (2017).

[62] Chang R. (2011). Practitioner reflections on engineering students' engagement with e-learning. Adv Eng Educ J.

[63] Deering J. (2016).

[64] Flyvbjerg B. (2006). Five misunderstandings about case-study research. Qual. Inq..

[65] Brindley C., Oxborrow L. (2014). Aligning the sustainable supply chain to green marketing needs: a case study. Ind. Market. Manag..

[66] Hupponen M., Grönman K., Horttanainen M. (2015). How should greenhouse gas emissions be taken into account in the decision making of municipal solid waste management procurements? A case study of the South Karelia region, Finland. Waste Manage. (Tucson, Ariz.).

[67] Campus G., UCC (2016). UCC Sustainability Strategy.

[68] UCC (2016).

[69] UCC (2020).

[70] Nardon L., Hari A., Aarma K. (2021). Reflective interviewing—increasing social impact through research. Int. J. Qual. Methods.

[71] Campbell S. (2020). *Purposive sampling: complex or simple? Research case examples.* J. Res. Nurs..

[72] Könnölä T. (2012). Facing the future: scanning, synthesizing and sense-making in horizon scanning. Sci public policy.

[73] Amanatidou E. (2012). On concepts and methods in horizon scanning: lessons from initiating policy dialogues on emerging issues. Sci. Publ. Pol..

[74] Tsakalidis A. (2021). Horizon scanning for transport research and innovation governance: a European perspective. Transp. Res. Interdiscip. Perspect..

[75] Braun V., Clarke V. (2006). Using thematic analysis in psychology. Qual. Res. Psychol..

[76] Creswell J.W., Creswell J.D. (2017). Sage publications. Research design: Qualitative, quantitative, and mixed methods approaches.

[77] Min M., Anderson J.A., Chen M. (2017). What do we know about full-service community schools? Integrative research review with NVivo. Sch. Community J..

[78] (2022). UCC. Sustainable Procurement.

[79] Baricco M. (2018). University of Turin performance in UI GreenMetric Energy and Climate Change.

[80] UCC. UCC "Saver Saves" Scheme (2022). https://www.ucc.ie/en/greencampus/practice/energy-water-and-climate-change/energy-management-at-ucc/.

[81] UCC. Sustainable Print Management Policy (2019). https://www.ucc.ie/en/procurement/news/sustainable-print-management-policy.html.

[82] (2019). DCCAE. Climate Action Plan.

[83] Kuriakose J. (2022). What does the Paris climate change agreement mean for local policy? Downscaling the remaining global carbon budget to sub-national areas. J Renew Sustain Energy Trans.

[84] Lee P., Lam P.T.I., Lee W.L. (2015). Risks in energy performance contracting (EPC) projects. Energy Build..

[85] Rolfe G., Freshwater D., Jasper M. (2001). Critical reflection for nursing and the helping professions: A user's guide..

[86] Borton T. (1970).

[87] Bryant I., Johnston R., Usher R. (2004).

